# Supplementary material for: Mesoscopic description of hippocampal replay and metastability in spiking neural networks with short-term plasticity
Source: PLoS Comput Biol. 2022 Dec 22;18(12):e1010809. doi: 10.1371/journal.pcbi.1010809 (PMC9822116; doi:10.1371/journal.pcbi.1010809)
Supplement: S1 Text — Fig A: shows “Fatigue-induced hippocampal replay in the macroscopic and in the Romani-Tsodyks model” in correspondence to finite-size-induced replay shown in Fig 4. Fig B: shows that “Up-Down dynamics depend on network size” and complements the discussion around Fig 3. Table A: provides a comparison between the simulation results for “Fatigue-induced hippocampal replay dynamics” in the micro-, meso- and macroscopic models. Table B: provides a comparison between the simulation results for “Finite-size-induced hippocampal replay dynamics” in the micro- and mesoscopic models. (PDF) [file pcbi.1010809.s001.pdf]

S1 Text — Supporting Information

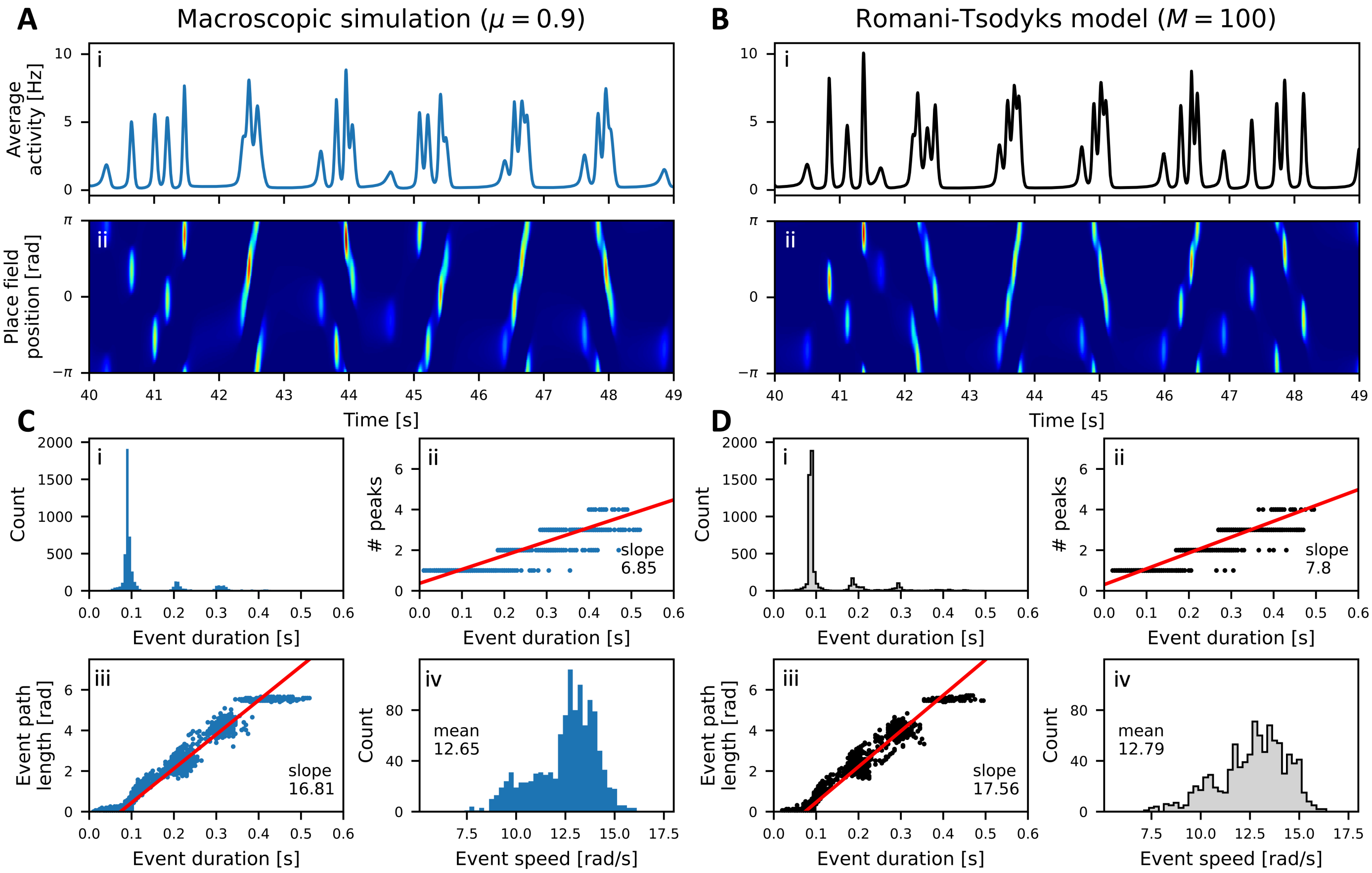

Fig A. Fatigue-induced hippocampal replay in the macroscopic and in the Romani-Tsodyks model. Simulation results of the deterministic (macroscopic and Romani-Tsodyks) models. Panels correspond to those in Fig 4. For a statistical comparison with the micro- and mesoscopic ring network models in the fatigue-induced regime of hippocampal replay dynamics, see Table A below.

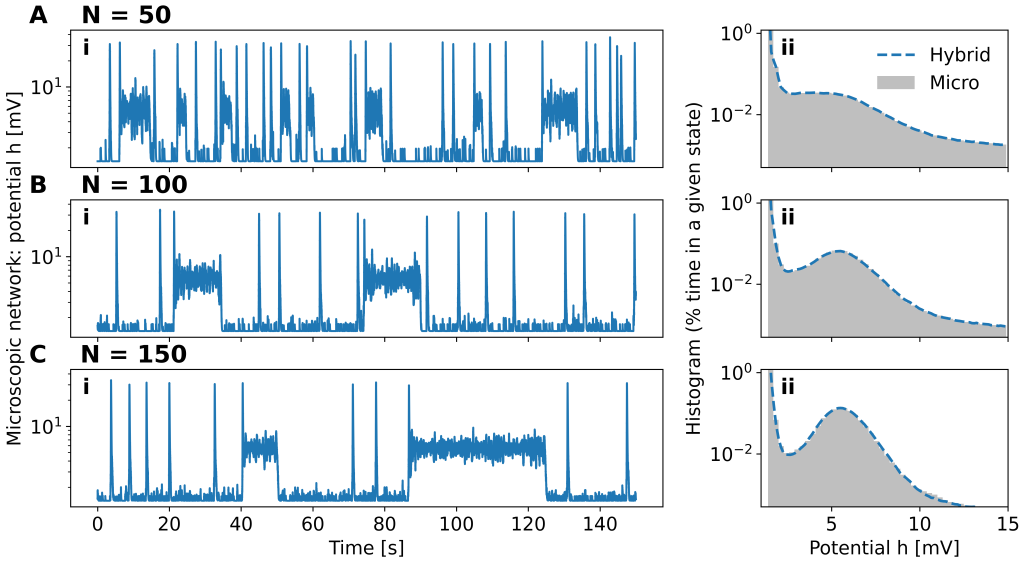

Fig B. Up-Down dynamics depend on network size. Finite-size effects on the Up-Down dynamics in a network of (A) $N = 50$, (B) $N = 100$, (C) $N = 150$ LNP neurons with STD. Column i: Trajectories of the input potential $h(t)$ for the microscopic network Eq. (1) with logarithmic y-scale with the same parameters as in Fig 3. The larger the network size, the longer the Up states and the less frequent population spikes. Column ii: Histogram of the potential $h(t)$ shows excellent agreement between microscopic (black) and mesoscopic dynamics (blue; Eq. (4)). Simulation length $T_{\text{sim}} = 10'000$ s.

Table A. Fatigue-induced hippocampal replay dynamics ($\mu = -0.9$).
Simulation results complementing Figs 4, 5 and A using the macroscopic ring network model (obtained from Eq. (8) in the limit $N \to \infty$) and the deterministic RT model. For comparison, we also report simulation results for finite-sized populations ($N = 50$) using the microscopic ring network Eq. (7), the mesoscopic jump-diffusion ring network model Eq. (8) with hybrid noise and the diffusion model Eq. (36) with Gaussian noise.

Common input: $\mu = -0.9$	Microscopic ($N = 50$)	Mesoscopic ($N = 50$) Jump-diff.	Mesoscopic ($N = 50$) Diffusion	Macroscopic ($N \to \infty$)	RT-model ($N \to \infty$)
T_{sim} [s]	1950	1950	1950	2350	2350
# bursts	5205	5172	5296	5167	5096
slope(# peaks/duration)	11.27	11.29	11.07	6.85	7.80
slope(distance/duration)	15.39	15.34	15.33	16.81	17.56
mean(IBI)	0.250	0.252	0.245	0.316	0.293
CV(IBI)	0.734	0.741	0.723	0.847	0.794
skewness γ_s(IBI)	1.312	1.357	1.373	1.224	1.230
resc. skewness α_s(IBI)	0.596	0.611	0.633	0.481	0.516
kurtosis γ_e(IBI)	1.620	1.828	2.036	0.454	0.499
resc. kurtosis α_e(IBI)	0.201	0.222	0.260	0.042	0.053
# NLE (> 1 peak)	1208	1232	1228	1140	939
fraction(NLE/bursts)	23.2%	23.8%	23.2%	22.1%	18.4%
fraction(forward/NLE)	51.1%	49.8%	50.2%	49.4%	51.5%
mean(abs(NLE speed))	10.07	9.96	9.73	12.64	12.79
Serial correlations					
Lag 1 (event speed)	0.086	0.086	0.035	-0.344	-0.563
Lag 1 (forward/backward)	0.132	0.138	0.096	-0.331	-0.525
Lag 2 (event speed)	0.019	0.027	0.017	0.229	0.434
Lag 2 (forward/backward)	0.026	0.029	0.044	0.226	0.401
Lag 3 (event speed)	0.032	-0.023	0.015	-0.074	-0.320
Lag 3 (forward/backward)	0.043	-0.019	0.017	-0.082	-0.312
Lag 4 (event speed)	0.015	0.014	-0.033	0.021	0.227
Lag 4 (forward/backward)	-0.004	-0.013	-0.051	0.024	0.215
Lag 5 (event speed)	0.022	-0.100	0.010	0.058	-0.139
Lag 5 (forward/backward)	0.007	-0.100	0.014	0.052	-0.135

Table B. Finite-size-induced hippocampal replay dynamics ($\mu = -1.4$).
Simulation results complementing Figs 4 and 5, see also Table 2, using the microscopic ring network Eq. (7), the mesoscopic jump-diffusion ring network model Eq. (8) with hybrid noise and the diffusion model Eq. (36) with Gaussian noise. The macroscopic ($N \to \infty$) and RT models remain in a globally quiescent state and do not exhibit metastable dynamics for the chosen parameters.

Common input:	Microscopic	Mesoscopic ($N = 50$)		Macroscopic	RT-model
$\mu = -1.4$	($N = 50$)	Jump-diff.	Diffusion	($N \to \infty$)	($N \to \infty$)
T_{sim} [s]	1950	1950	1950	2350	2350
# bursts	5030	5040	5135	—	—
slope(# peaks/duration)	9.28	9.26	9.56	—	—
slope(distance/duration)	17.27	17.17	17.09	—	—
mean(IBI)	0.652	0.651	0.636	—	—
CV(IBI)	0.846	0.842	0.817	—	—
skewness γ_s(IBI)	0.917	0.940	0.900	—	—
resc. skewness α_s(IBI)	0.361	0.372	0.367	—	—
kurtosis γ_e(IBI)	-0.115	-0.047	-0.097	—	—
resc. kurtosis α_e(IBI)	-0.011	-0.004	-0.010	—	—
# NLE (> 1 peak)	1019	967	1025	—	—
fraction(NLE/bursts)	20.3%	19.2%	20.0%	—	—
fraction(forward/NLE)	51.0%	47.9%	49.1%	—	—
mean(abs(NLE speed))	12.41	12.54	12.46	—	—
Serial correlations					
Lag 1 (event speed)	0.048	0.054	0.034	—	—
Lag 1 (forward/backward)	0.062	0.062	0.033	—	—
Lag 2 (event speed)	-0.043	-0.010	0.043	—	—
Lag 2 (forward/backward)	-0.041	-0.001	0.040	—	—
Lag 3 (event speed)	-0.013	0.053	-0.055	—	—
Lag 3 (forward/backward)	-0.018	0.056	-0.051	—	—
Lag 4 (event speed)	-0.010	0.011	0.007	—	—
Lag 4 (forward/backward)	-0.015	0.007	0.001	—	—
Lag 5 (event speed)	0.008	-0.032	0.032	—	—
Lag 5 (forward/backward)	0.007	-0.033	0.035	—	—
