# Supplementary material for: Mesoscopic description of hippocampal replay and metastability in spiking neural networks with short-term plasticity
Source: PLoS Comput Biol. 2022 Dec 22;18(12):e1010809. doi: 10.1371/journal.pcbi.1010809 (PMC9822116; doi:10.1371/journal.pcbi.1010809)
Supplement: Attachment — Submitted filename: reportPLOS.pdf [file pcbi.1010809.s002.pdf]

The authors analyze stochastic mean-field models developed for ensembles of simple Poissonian neurons with short-term depression, where the neurons are driven by a common field encompassing the single neuron plasticity which evolves driven by the corresponding Poissonian spike train. In particular, the authors mostly consider finite size networks of leaky integrate-and-fire neurons. The authors apply their mean field formulation, encompassing finite size effects, in a few cases to show the relevance of finite size fluctuations for the occurrence of population bursts, up and down dynamics and for a model of hippocampal replay dynamics.

I find the reported results interesting and worth of publication, however the mathematical jargon employed by the authors and the non clear definition of the employed models risk to render quite hard the reading of the manuscript for non mathematicians. I suggest to the author to largely rewrite the manuscript trying to render the presentation of the employed models more readable for a non specialized audience.

Let me now be more specific a list a series of comments/remarks that the authors should address :

1) The authors in the Introduction wrote :

"Mean-field models of STP [23] have recently gained renewed attention [37, 38] in the context of the Montbrió-Pazó-Roxin theory for quadratic integrate-and-fire neurons [39, 40]. However, in these models the mean-field description of STP is heuristic – it is not derived from a microscopic model but introduced ad hoc at the population level. More importantly, the models are deterministic corresponding to the limit of infinitely many neurons, and thus cannot explain fluctuation-induced transitions among metastable states in finite-size networks."

The introduction of the STP in the Montbrió-Pazó-Roxin has been done at a mean-field level by neglecting fluctuations, not in a heuristic manner, this corresponds to the model of the authors (3) when the variable Q is neglected, i.e. in the thermodynamic limit N --→ infinite, as expected for a mean field model. Furthermore, the models studied in [37,38,39,40] refers to exact derivation of mean field dynamics of recurrent spiking heterogeneous networks, quite far from a heuristic mean field model. The model presented by the authors is instead based on heuristic transfer functions (see eq (5)). Therefore, I suggest to the authors to rewrite this sentence in a more correct way.

Finally I would like to stress that Lorentzian and Gaussian fluctuations (finite size or of any other kind) has been recently introduced in the Montbrió-Pazó-Roxin formulation, see :

D. Goldobin, et al. Ph*ysical Review Letters* 127,038301 (2021)

Clusella, P., & Montbrió, E. (2022). Regular and sparse neuronal synchronization are described by identical mean field dynamics. *arXiv preprint arXiv:2208.05515*.

2) The definition of the microscopic model (1) is completely unclear to me. The authors should specify:

I) If the model is a spiking model or not. Are the authors just describing sub-threshold dynamics ?
II) What do they mean by Pois[f(h_i(t^-))dt].
III) How are generated exactly the Poissonian spike trains "delivered" by each neuron ?
IV) The function f() is not defined at this stage, and it should. I guess f(h) is the firing rate but it should be clarified.
V) What do the authors mean by exponential impulse response function ? Is this the one reported in Eq (5) ? Is this the one for the leaky integrate-and-fire neuron ?

VI) What the authors mean by conditional intensities ?

I have not found these information nor in the main text neither in the Methods, and these are fundamental details to understand the model.

3) Page 5, just figure 1, I guess there is a misprint:

“The trajectories h_i(t) are cadlag …..” (?)

4) In Equations (3) it appears a function f() which has not been yet defined, as in Eq. (2).

5) Eqs (3) and (4) are stochastic differential equations (SDEs) with multiplicative noise terms, the authors should clarify in details in the Methods how these equations are numerically integrated. Which integration scheme is employed ? Are the authors performing the integration of these SDEs in the Stratanovich or Ito sense ? Why do they choose Ito’s or Stratanovihc’s formulation ? Please report explicitly the integration procedure, since this is extremely delicate for a SDE with multiplicative noise.

6) At the end of page 6 the authors affirm that for sufficiently large N they can rewrite also the shot-noise term as average term plus Gaussian noise \xi_a and that this noise term is independent by the Gaussian noise \xi_x. This is not clear for me, since the 2 stochastic processes are associated to the neural activity of the same neuronal ensemble, may the authors better clarify this issue ?

7) Transfer function in equation (5) – It is not clear for me how the transfer function reported in Eq (5) is defined. Please, may the authors clarify the following points even in the Methods or in the text :

I) How is defined the threshold on (5), what happens when the membrane potential overcomes the threshold ? Is the membrane potential reset to same value ? Is a spike sent to the neurons ?
II) Why do the authors choose such kind of transfer function ? Is there some biological reason for that ?

8) The authors wrote in page 7 :

“These bursts of activity, called population spikes, have been studied theoretically in the context of STP [41, 51–53] and have also been observed experimentally [53, 54]. Here, we complement the existing literature by pinpointing to the (finite) network size as a possible mechanism for endogenously generated population spikes without the need for external (noisy) inputs.”

I think this affirmation is somehow misleading, since the model studied in [53] is fully deterministic, no external noise, however the network is random (not fully coupled as the one studeied by the authors) and therefore endogeneous fluctuations emerge spontaneously in the system and they can be the origin of the observed bursts, indeed they are. May the authors clarify better which is there contribution ? Since endogenously generated population spikes in fully deterministic systems have been already studied in [51] ?

9) The authors should clearly define what is a macroscopic model for them in the first pages, I have only realized at page 7 or 8 that by macroscopic model they mean the mean-field model without finite size fluctuations.

10) In page 30 , the authors wrote :

“ Next, we defined nonlocal replay events (NLEs) as bursts of the average activity with more than one peak so that a transient traveling wave is visible in the density plots in Fig. 4Bii and Cii, resembling a hippocampal replay pattern. Among all bursts events, around 20% are NLEs, which is consistent across all four models. Around half of all the NLEs travel in anti-clockwise/negative direction (“forward replay”) and the other half in clockwise/positive direction (“backward replay” or “preplay”); also this feature is consistent across the different models. “

Is this feature observable also in experiments ? If yes please provide references. In particular, in experiments is possible to observe forward and backward replays ? Or just backward ones ?

In summary I regret to say that at this stage the manuscript is not suitable for publication in PLOS Computational Biology and a large rewriting is required before resubmitting the amended manuscript.
