# Supplementary material for: Mesoscopic description of hippocampal replay and metastability in spiking neural networks with short-term plasticity
Source: PLoS Comput Biol. 2022 Dec 22;18(12):e1010809. doi: 10.1371/journal.pcbi.1010809 (PMC9822116; doi:10.1371/journal.pcbi.1010809)
Supplement: Attachment — Submitted filename: response_letter.pdf [file pcbi.1010809.s003.pdf]

Dear Editor,

We truly appreciate the feedback on our manuscript. We are glad that the referees consider our work interesting, clear, well-written, and potentially important. We did our best to incorporate their advice and amended our manuscript accordingly. We believe that we have fully addressed all comments made by the referees. Please find a summary of changes below. Next to our point-by-point response to the comments, we attached a pdf version of the revised manuscript, in which all adjustments have been highlighted in red.

Best regards,

Bastian Pietras, Valentin Schmutz and Tilo Schwalger

List of changes

- We improved the presentation of the microscopic LNP-STD model for single (around Eq. (1)) and multiple populations (around Eq. (8)) with detailed explanations of the technical aspects of the model.
- Despite a more careful presentation of the microscopic model and an additional subsection on the macroscopic model, we shortened the results section by 1.5 pages. We believe that the manuscript is now better streamlined as large parts are now moved to the Discussion.
- The mesoscopic ring-network model Eq. (8) takes now on the form of the jump-diffusion model with hybrid noise instead of the simplified diffusion model. This change acknowledges the importance of accurately capturing the properties of finite-size fluctuations that underlie the novel regime of finite-size induced hippocampal replay.
- We restructured the Discussion, where we included three new subsections (mainly taken from the Results part):
  Diffusion- vs./ jump-diffusion model: it depends on network size and dynamical regime;
  Network size variation resembles long-term plasticity;
  Modelling hippocampal replay.

Response to First Referee

Referee: The authors introduce a theoretical framework that allows one to derive the dynamics of homogeneous but finite-size neuronal populations, in presence of short-term synaptic depression. The "finite-sizeness" (if I can say so) turns out to be important, because it results in the statistics of the "noise" at the mesoscopic level, which is rigorously derived from the microscopic description. This is to be contrasted with standard approaches where the noise (typically white) is added on a phenomenological basis. The framework is then applied to two different models (a model of up-down dynamics and a model of hippocampal-replay dynamics) and it is shown to be very accurate from a quantitative point of view. In both cases, the impact of finite-size noise is carefully studied.

The manuscript contains interesting results, mostly from a theoretical/mathematical point of view, but the numerical simulations presented could (should) be of interest also for more experimentally-oriented readers. The results are novel as far as I can say. The manuscript also includes a lucid discussion of the limitations of the presented results. Overall, it is clearly written though, perhaps, a little too long.

I recommend publication.

Response:
We thank the Referee very much for taking their time to read and assess our manuscript by pointing out that it contains interesting results both for theoretically- and experimentally-oriented readers. We are happy that she/he supports publication of our manuscript and we hope that she/he will still do so even after taking into account the many amendments we have made to streamline our manuscript better and in response to the other two Referees' comments.

Response to Second Referee

Referee: The authors analyze stochastic mean-field models developed for ensembles of simple Poissonian neurons with short-term depression, where the neurons are driven by a common field encompassing the single neuron plasticity which evolves driven by the corresponding Poissonian spike train. In particular, the authors mostly consider finite size networks of leaky integrate-and-fire neurons. The authors apply their mean field formulation, encompassing finite size effects, in a few cases to show the relevance of finite size fluctuations for the occurrence of population bursts, up and down dynamics and for a model of hippocampal replay dynamics.

I find the reported results interesting and worth of publication, however the mathematical jargon employed by the authors and the non clear definition of the employed models risk to render quite hard the reading of the manuscript for non mathematicians. I suggest to the author to largely rewrite the manuscript trying to render the presentation of the employed models more readable for a non specialized audience.

Response:
We thank the referee for the detailed assessment of our manuscript and the important remark that the model might be difficult to understand for some readers. We apologize for not being clear enough with the definition of the model. For example, we do not use integrate-and-fire neuron models but a different class of commonly used spiking neuron models and this might also be the source of some confusion. The criticism is highly useful because it shows us which parts of the model description could be capable of being misunderstood.
We have now rewritten the presentation of the model according to the suggestions by the referee (see points below). We also use less technical jargon and explain all notations. We hope that the new presentation has improved and that the model is now comprehensible to a broad readership.

Referee: Let me now be more specific a list a series of comments/remarks that the authors should address:

1) The authors in the Introduction wrote:

"Mean-field models of STP [23] have recently gained . . . in finite-size networks."

The introduction of the STP in the Montbrió-Pazó-Roxin has been done at a mean-field level by neglecting fluctuations, not in a heuristic manner, this corresponds to the model of the authors (3) when the variable Q is neglected, i.e. in the thermodynamic limit $N \to \infty$, as expected for a mean field model. Furthermore, the models studied in [37,38,39,40] refers to exact derivation of mean field dynamics of recurrent spiking heterogeneous networks, quite far from a heuristic mean field model. The model presented by the authors is instead based on heuristic transfer functions (see eq (5)). Therefore, I suggest to the authors to rewrite this sentence in a more correct way.

Finally I would like to stress that Lorentzian and Gaussian fluctuations (finite size or of any other kind) has been recently introduced in the Montbrió-Pazó-Roxin formulation, see:

D. Goldobin, et al. Physical Review Letters 127,038301 (2021)
Clusella, P., & Montbrió, E. (2022). Regular and sparse neuronal synchronization are described by identical mean field dynamics. arXiv preprint arXiv:2208.05515.

Response:
We thank the referee for bringing up this point and use this chance to clarify what we mean by "heuristic". In short, we agree that the wording may be confusing so we have now tried to avoid the word "heuristic". The part now reads:

Mean-field models of STP [23] have recently gained renewed attention [37,38] in the context of the Montbrió-Pazó-Roxin theory for quadratic integrate-and-fire neurons [39,40]. These macroscopic models are exact in the case of static synapses, or when STP is introduced at the population level, but not when dynamic synapses undergo STP individually. Moreover, the resulting firing rate equations are deterministic—as they hold in the limit of infinitely many neurons independently of individual neurons being subject to Gaussian [41] or Cauchy noise [42,43]—and thus cannot explain fluctuation-induced transitions between metastable states in finite-size networks.

To provide more details, in references [37,38] low-dimensional mean-field models are only derived under the (heuristic) assumption that the spike trains emitted by each neuron are Poissonian and that, therefore, the individual synapses can be approximated by a mesoscopic description (Eq.(11) in [37]). The dynamics of the quadratic integrate-and-fire network with such mesoscopic STP can be reduced along the Montbrió-Pazó-Roxin theory. In general, however, the Poisson assumption does not hold for quadratic integrate-and-fire neurons when they are globally coupled (as required by the Montbrió-Pazó-Roxin theory). By contrast, the Poisson assumption is already hardwired in our microscopic model, which enables us to derive mesoscopic mean-field models that are also valid for finite network sizes.
Besides, we thank the referee for pointing us to the work by Goldobin et al. and by Clusella et al., see also the recent preprint by Pietras et al. (arXiv:2209.00922). Note, however, that even in presence of microscopic Gaussian or Cauchy fluctuations the resulting macroscopic mean-field models are deterministic. As of now, an extension to incorporate finite-size fluctuations has proven elusive.

Referee: 2) The definition of the microscopic model (1) is completely unclear to me. The authors should specify:

I) If the model is a spiking model or not. Are the authors just describing sub-threshold dynamics?

II) What do they mean by Pois[$f(h_i(t^-))dt$].

III) How are generated exactly the Poissonian spike trains "delivered" by each neuron ?

IV) The function f() is not defined at this stage, and it should. I guess f(h) is the firing rate but it should be clarified.

V) What do the authors mean by exponential impulse response function ? Is this the one reported in Eq (5) ? Is this the one for the leaky integrate-and-fire neuron ?

VI) What the authors mean by conditional intensities ?

I have not found these information nor in the main text neither in the Methods, and these are fundamental details to understand the model.

Response: We agree that the presentation of the microscopic model was unclear on several points and we have completely rewritten the presentation. The new formulation of the model should be unambiguous and accessible to a broader audience. Here are our point-by-point responses:

I) The model is a spiking model since neurons interact via spikes. Moreover, Linear-Nonlinear-Poisson neurons (nonlinear Hawkes processes, or simply, Poisson neurons) are relatively well-established spiking neuron models [Burak and Fiete, 2012, Gerstner et al., 2014, Hennequin et al., 2018, Brinkman et al., 2018]. The confusion might have come from the fact that, unlike standard *threshold-based* models (e.g., leaky integrate-and-fire neurons), Linear-Nonlinear-Poisson (LNP) neurons do not reset their potential $h_i(t)$ after each spike and they do not have a hard threshold. We hope that the new presentation of the model makes it clear that the model is indeed a (stochastically) spiking model.

II) $\text{Pois}[f(h_i(t^-))dt]$ denotes the law of a Poisson random variable with mean $f(h_i(t^-))dt$, in particular $dn_i(t) \sim \text{Pois}[f(h_i(t^-))dt]$ means that the number of spikes dn_i of neuron i in the time interval $[t, t+dt)$ is Poisson-distributed with mean $f(h_i(t^-))dt$. Note that when dt is small, $\text{Pois}[f(h_i(t^-))dt]$ has approximately the same law as a Bernoulli random variable with parameter $f(h_i(t^-))dt$, i.e. $dn_i(t)$ can only be either 0 (no spike) or 1 (spike). (The notation t^- means the time just before t.) This notation was indeed ambiguous in the context of the microscopic model; we have replaced it with a more transparent definition.

III) The new presentation of the model gives a much more concrete and constructive definition of the stochastic spike trains which is very close to the numerical implementation of the microscopic model. We now write: **"In numerical simulations of networks of N LNP neurons, in each small time step $[t, t+\Delta t)$, we draw N independent Bernoulli random variables $z_{t,1}, \dots, z_{t,N} \in \{0,1\}$ with success probabilities $f(h_1(t^-))\Delta t$, ..., $f(h_N(t^-))\Delta t$, respectively. If $z_{t,i} = 1$, then neuron i emits a spike at time t."**

IV) The function $f()$ was indeed not defined. We thank the reviewer for noting this. We have corrected this omission and refer to f as the static nonlinearity of the LNP model. We first define f as a general non-negative increasing function in the Theory part, and then specify the concrete choice of f used in simulations when we compare microscopic and mesoscopic simulations.

V) By "exponential impulse response function", we meant a form of the linear filter of the LNP model and we did not mean the static nonlinearity $f()$. When the linear filter of the LNP is an "exponential impulse response function", the potential $h_i(t)$ is simply a leaky integrator of the total input the neuron receives (see Eq. (1)). In the current manuscript, we have omitted this somewhat misleading expression.

VI) By conditional intensities, we mean the instantaneous hazard rates $f(h_i(t^-))$ at which the LNP neurons emit spikes depending on their current input potentials h_i. We have clarified the definition of the conditional intensity / hazard rate in the presentation of the microscopic model and explicitly state the conditional probability for neuron i to elicit a spike within a short time interval $[t, t+\Delta t)$ given its actual input potential $h_i(t^-)$ as

$$\text{Prob}\{\text{spike of neuron } i \text{ in } [t, t+dt) \big| h_i(t^-)\} = f(h_i(t^-))dt,$$

which implicitly defines the conditional intensity.

Referee: 3) Page 5, just figure 1, I guess there is a misprint:
"The trajectories $h_i(t)$ are cadlag ....." (?)

Response: We have replaced the technical term "cadlag" with the more understandable term "right-continuous".

Referee: 4) In Equations (3) it appears a function f() which has not been yet defined, as in Eq. (2).

Response:
The function f is now clearly introduced around Eq. (1) as a non-negative increasing function that represents the static nonlinearity of the LNP model, i.e. it determines the instantaneous firing rate (conditional intensity) of individual neurons. The concrete functional form used in simulations is provided in the corresponding section, where we compare microscopic vs. mesoscopic simulations. In Eq.(3), the function f obtains a "new" mesoscopic role. The link between the microscopic f in Eq.(1a) and its interpretation for mesoscopic dynamics is now made explicit in a new section on the macroscopic mean-field model, which hopefully clarifies previous concerns about the interpretation of the function f. Please, also see our answer to point 2(IV), above, and point 7, below.

Referee: 5) Eqs (3) and (4) are stochastic differential equations (SDEs) with multiplicative noise terms, the authors should clarify in details in the Methods how these equations are numerically integrated. Which integration scheme is employed ? Are the authors performing the integration of these SDEs in the Stratanovich or Ito sense ? Why do they choose Ito's or Stratanovich's formulation ? Please report explicitly the integration procedure, since this is extremely delicate for a SDE with multiplicative noise.

Response:
It is true what we should have explicitly written the all SDEs are integrated in the sense of Itô. Just after Eq. (3), we now write:

> **"The stochastic differential equation (SDE) (3), as all the SDEs in this work, is integrated in the sense of Itô. Numerically, this means that for each integration time step, the factor $\sqrt{Qf(h)/N}$ multiplying the noise is evaluated at the time *just before* the random increment."**

We use Itô integration of the Gaussian white noise because it derives from the Itô integration of the Poisson noise in the microscopic model (we have now clarified in the presentation of the microscopic model (1) that the system of SDEs is integrated in the sense of Itô).
All integration schemes are straightforward Itô numerical integrations and can be obtained from Eq. (19) in the Methods. We do not explicitly write the numerical integration schemes for all the models considered in this work, as this would unnecessarily lengthen the present manuscript.

Referee: 6) At the end of page 6 the authors affirm that for sufficiently large N they can rewrite also the shot- noise term as average term plus Gaussian noise ξ_a and that this noise term is independent by the Gaussian noise ξ_x. This is not clear for me, since the 2 stochastic processes are associated to the neural activity of the same neuronal ensemble, may the authors better clarify this issue ?

Response:
We have removed the sentences involving the noise ξ_A on page 6; the noise ξ_A now only appears in the Methods section "Reduction of the jump diffusion model to the pure diffusion process". The explanation for why the noises ξ_A and ξ_x are independent is nontrivial, and can be found in all detail in Schmutz, Gerstner and Schwalger (2020). The fact that the explanation is rather complicated was one of the main motivations for the derivation of the pure diffusion model (3), which has a unique source of noise ξ. As mentioned in the main text, the derivation of Eq. (3) is much more straightforward and transparent than the derivation of the original model of Schmutz, Gerstner and Schwalger (2020).

Referee: 7) Transfer function in equation (5) – It is not clear for me how the transfer function reported in Eq (5) is defined. Please, may the authors clarify the following points even in the Methods or in the text :

I) How is defined the threshold on (5), what happens when the membrane potential overcomes the threshold ? Is the membrane potential reset to same value ? Is a spike sent to the neurons ?

II) Why do the authors choose such kind of transfer function ? Is there some biological reason for that ?

Response:
We thank the referee for pointing out that the definition of the transfer function may be unclear and for pointing us to possible confusions of the reader. First, we would like to clarify the definition of the "transfer function" f. In our bottom-up framework, the function $f(h)$ is defined as the static nonlinearity of the LNP model (Linear-Nonlinear-Poisson cascade model) at the microscopic (neuronal) level. It turns out that at the meso- and macroscopic level, this static nonlinearity

becomes the nonlinear function of classical population-rate models (Wilson-Cowan-type models), in which case the nonlinear function is sometimes called transfer function. Put differently, the nonlinear function that emerges in the macroscopic model has a microscopic interpretation (namely the static nonlinearity of the LNP model). To emphasize that the function f in the meso- and macroscopic models is not a free functions but is determined by the microscopic model, we now refer to f only as the static nonlinearity of the LNP model and avoid the use of the imprecise term "transfer function" throughout the revised manuscript. Specifically, we now write

> **[...] where we identified $A(t) = f(h(t))$ by taking the limit $N \to \infty$ in Eq. (4c). Thus, the transfer function that relates the input potential $h(t)$ to the neuronal population activity $A(t)$ in the macroscopic model is exactly given by the static nonlinearity f of the LNP neuron model, Eq. (1). Put differently, the nonlinear function f in the macroscopic model has a precise microscopic interpretation as the static nonlinearity of the microscopic model.**

Let us now address the specific points raised by the referee:

I) In contrast to integrate-and-fire models, in the LNP model there is no sharp threshold but a soft threshold in form of a firing intensity that determines the probablity to fire depending on the input potential $h(t)$. Roughly speaking, below the "threshold" h_0 the firing probablity is small and it is rapidly increasing around h_0. Well above the threshold the firing probability is high. In the limit where the softness parameter a goes to 0, the nonlinear function converges to a rectified linear function and the firing probability strictly vanishes when $h < h_0$. In a simulation, in each small time step of size Δt, a spike is emitted with probability $f(h(t))\Delta t$. If a spike occurs, it is send to all neurons in the network because we consider full connectivity. The input potential remains unaltered, i.e. it is not reset upon spiking. Thus, the LNP model does not account for post-spike refractoriness.

II) The specific static nonlinearity (transfer function) $f(h)$ has been chosen in correspondance with previous models [Holcman and Tsodyks, 2006, Romani and Tsodyks, 2015] in order to enable comparison between our and their work. This choice is also consistent with theoretical considerations [Aviel and Gerstner, 2006]. In general, a biologically plausible transfer function in firing-rate models should be an increasing, non-negative function of the input, as is in our case. Often it also exhibits saturation for large inputs giving rise to a sigmoidal function and preventing unbounded growth of firing rates. However, in the presence of synaptic depression an explicit saturation of the transfer function is not needed because unbounded growth of firing rates does not occur with our linearly increasing transfer function. We mentioned that transfer functions are often heuristic and chosen as simple as possible, e.g. [Hennequin et al., 2018], but they can also be fitted to experimental data (e.g. f-I curves) [e.g. Lim et al., Nat. Neurosci. (2015)] or chosen as the f-I curve of biophysical neuron models, e.g. [Ostojic and Brunel, 2011].

Referee: 8) The authors wrote in page 7 :
"These bursts of activity, called population spikes, have been studied theoretically in the context of STP [41, 51–53] and have also been observed experimentally [53, 54]. Here, we complement the existing literature by pinpointing to the (finite) network size as a possible mechanism for endogenously generated population spikes without the need for external (noisy) inputs."
I think this affirmation is somehow misleading, since the model studied in [53] is fully deterministic, no external noise, however the network is random (not fully coupled as the one studeied by the authors) and therefore endogeneous fluctuations emerge spontaneously in the system and they can be the origin of the observed bursts, indeed they are. May the authors clarify better which is there contribution ? Since endogenously generated population spikes in fully deterministic systems have been already studied in [51] ?

Response:
We thank the referee for his/her suggestion to clarify the relation to previous work [51,53] on population spikes. We agree that endogeneously generated population spikes in finite-size networks have been studied before, and that our sentence could be misunderstood. Accordingly, we have removed the sentence "Here, we complement the existing literature by pinpointing to the (finite) network size as a possible mechanism for endogenously generated population spikes without the need for external (noisy) inputs.".
Our novel contribution is the multi-scale description of emerging population spikes comprising both a microscopic network model and a corresponding mesoscopic model that accurately reproduces population spike activity, links uniquely to the parameters of the microscopic model but is much simpler and hence permits a mathematical analysis (e.g. phaseplane analysis). Thus, the mesoscopic model enables a theoretical understanding of the mechanisms of emerging dynamics (e.g. whether population spikes are fluctuation-induced or fatigue-induced). Since the mesoscopic model has no free parameters, each parameter of that model has a clear microscopic interpretation and as such might be constrained to physiological ranges or by measurements. It also allows an efficient analysis of the parameter dependence of population spikes, e.g. network size, which has a strong influence and is computationally expensive to study with a microscopic model (esp. the limit of large networks). Conversely, the mesoscopic model linked to microscopic parameters may be used for efficient parameter estimation, given mesoscopic data. Therefore, we believe that having a model of metastability in spiking neural networks at two different levels of granularity (microscopic and mesoscopic) is already highly valuable, and in particular may be useful to better understand population spikes in experiments and spiking neural network simulations. We note that the previous studies [51,53], considered either mesoscopic [53] or microscopic [51] models but did not present models on both scales that quantitatively matched each other.

Referee: 9) The authors should clearly define what is a macroscopic model for them in the first pages, I have only realized at page 7 or 8 that by macroscopic model they mean the mean-field model without finite size fluctuations.

Response:
We thank the referee for noting that the definition of "macroscopic model" was not introduced in the beginning. We have now added a corresponding sentence already in the introduction:

> **In the case of mean-field models, fluctuations are usually not described at all because they vanish in the mean-field limit of infinitely many neurons, $N \to \infty$. We will refer to such deterministic mean-field models as *macroscopic* models.**

This sentence should now also highlight the difference to *mesoscopic models*, which were already defined in the same paragraph as models with $N < \infty$ and noise.

Referee: 10) In page 30 , the authors wrote :
"Next, we defined nonlocal replay events (NLEs) as bursts of the average activity with more than one peak so that a transient traveling wave is visible in the density plots in Fig. 4Bii and Cii, resembling a hippocampal replay pattern. Among all bursts events, around 20% are NLEs, which is consistent across all four models. Around half of all the NLEs travel in anti-clockwise/negative direction ("forward replay") and the other half in clockwise/positive direction ("backward replay" or "preplay"); also this feature is consistent across the different models." Is this feature observable also in experiments ? If yes please provide references. In particular, in experiments is possible to observe forward and backward replays ? Or just backward ones ?

Response:
We thank the referee for their question and take this chance to make up for this missed chance to cite the relevant literature. Indeed, forward and backward replays are observed in the same animal within one experimental session, see the references [Davidson et al 2009, Gupta et al 2010].

Gupta et al. (Neuron 2010) report that "forward and backward replays were observed with similar proportion". Davidson et al. (Neuron 2009) likewise reported forward- and reverse-ordered replay within the same session of an animal, although with no clear trend about forward/backward directions in four tested rats (58%,51%,25%,21%). Note however that Davidson et al. tested rats exploring (and stopping along) a linear track. Here, we considered that the hypothetical animal had explored an idealized ring and therefore assume that the ratio between forward and backward replay is roughly 50%. More importantly, all models exhibit on average the same number of forward and of backward replay events and therefore allow a comparison between models.
We added the required citations at the respective part of the manuscript.

Referee: In summary I regret to say that at this stage the manuscript is not suitable for publication in PLOS Computational Biology and a large rewriting is required before resubmitting the amended manuscript.

Response:
We now have incorporated the Referee's valuable feedback and largely rewritten our manuscript, especially concerning the presentation of the microscopic model in a less mathematical jargon and suitable for a broader audience. We further hope to have answered all the Referee's concerns so that she/he shares our belief that the manuscript is suitable for publication in *PLoS Computational Biology*.

Response to Third Referee

Referee: The manuscript presents a derivation of a mesoscopic mean-field dynamics for a network of LNP-STD (linear-nonlinear Poisson with short-term plasticity/depression) spiking neurons capable to describe the evolution of the mean membrane potential and the mean fraction of available synaptic resources as in (Holcman and Tsodyks, 2006 – cited [HT06]). Resorting a temporal coarse-graining a la Gillespie, the authors incorporate a state- and size-dependent driving noise whose fluctuations are modulated by the size N, the second moment Q and the gain function f (the activity level). They further extend this mesoscopic description to the case in which the number of neurons is relatively small such that the assumptions underlying the diffusion approximation are broken, eventually deriving a hybrid-noise version of the mesoscopic dynamics. As application, the authors study hippocampal replay in a model network composed of such stochastic mesoscopic populations. The aim is to prove that differently to previous fatigue-driven deterministic models (Romani and Tsodyks, 2015 – cited [RT15]), on longer timescales they differ from the one introduced here as relying on fluctuation-driven metastability.
Overall I found this work interesting and sound, and surely it deserves to be published in PLoS Computational Biology. My feeling is that some parts of the paper appear to be not necessary, as detailed below together with a list of concerns to be addressed before the manuscript is published:

Response:
We thank the Referee very much for their support to have our manuscript published in *PLoS Computational Biology*. Given the Referee's very nice and precise summary of our results, we are optimistic that many more readers will agree that our work is interesting and sound, especially after having incorporated the highly valuable comments raised by this and the other Referees. We have streamlined our manuscript now better, also to make it more accessible for a wider audience. Our point-to-point answers to all the Referee's concerns including additional simulations can be found below.

Referee: 1. Why is the hybrid-noise version of the mesoscopic dynamics needed? At page 12 the authors write "In the following, we use the simpler diffusion model because it already faithfully reproduces microscopic simulations." So for a large part of this paper the mesoscopic dynamics (4) does not seem to be useful to the aims of the authors. Furthermore, I found some degree of contradiction in using the purely diffusive dynamics (3) for the mesoscopic model describing the hippocampal replay, where the size of the single populations is $N = 50$. This is exactly the range of neuron number presented in Fig. 2 ($N = 20$ vs $N = 200$) and Fig. 3 ($N = 100$) where the hybrid-noise version works better. One could interpret the chosen presentation of the results as a proof of the fact that, when at the multiscale level, the hybrid-noise system does not provide any quantitative advantage. If this is the case, why to present it in this paper?

Response:
We sincerely thank the Referee for raising this point as it allows us to resharpen and even strengthen our message. At the same time, we have to correct our previous statement that the simpler diffusion model can faithfully reproduce microscopic simulations. Indeed, in Figs. 4–6, we used the jump-diffusion model for simulations. We apologize for this mistake and, in the following, try to (i) explain why we wrote this sentence in the first place and (ii) clarify why the jump-diffusion model with hybrid noise is central to our paper. Despite our mistake in providing the equations of the wrong version of the mesoscopic ring network model, the results and implications of our paper remain unaltered. We now have stressed more clearly the importance of accurately describing finite-size fluctuations for emerging metastable network dynamics, and that both network size and dynamical regime under investigation should inform the choice of either the simpler diffusion model with Gaussian noise or the jump-diffusion model with hybrid noise.
To provide more details, as mentioned in the previous introduction to the Results section on hippocampal replay (large parts of this introduction are now moved to the Discussion, but see our

reply below), we realized the need for a bottom-up stochastic ring network model with short-term depression that can faithfully describe the spontaneously occurring NLE states, which Romani and Tsodyks in [RT15] attributed to hippocampal replay dynamics. Hence, we extended our one-population model to a ring network with multiple populations in a similar setup as in [RT15] and first corroborated their findings using a macroscopic ring network model in the limit of infinitely many neurons per population. We found an analogous deterministic regime of spontaneously emerging hippocampal replay patterns, see Fig. S2 in the Supplementary Material, where the NLE states are fatigue-induced. Subsequently, we tested our mesoscopic models by changing the network size in order to retrieve this dynamics both in mesoscopic and microscopic simulations. Indeed, for this regime of fatigue-induced hippocampal replay, the diffusion model with Gaussian noise already proved a very accurate description of the microscopic network dynamics with finite population sizes. Up to this stage, the presentation of the mesoscopic ring model Eq. (8) and the phrase quoted by the Referee is fully in order.
After that, however, we started investigating other parameter sets. In particular, we decreased the common input $\mu = -0.9 \mapsto -1.4$ and, suddenly, the microscopic network simulations featured hippocampal replay dynamics with spontaneously occurring NLEs for small population sizes $N = 50$, but not for large $N = 5000$, see Fig. 4. This is a dynamical regime of finite-size induced hippocampal replay, which could only be recovered with the mesoscopic ring network models. The jump-diffusion model Eq. (8) with hybrid noise proved here quantitatively advantageous over the diffusion model. Importantly, the macroscopic model was not able to recover the metastable dynamics at all and remained in a quiescent state.
In sum, the hybrid-noise version of the mesoscopic dynamics does indeed provide a quantitative advantage over the diffusion model and therefore deserves to be central in our paper. For this reason, we have changed the Results section "Microscopic and mesoscopic multi-population model of place cells", where we now present the mesoscopic jump-diffusion model with hybrid noise and mention in the text that the diffusion model can be readily reduced from the jump-diffusion model, see also the Methods section, where we provide the diffusion ring model in Eq. (36). We now also discuss in more detail in the Discussion section "Diffusion- vs. jump-diffusion model: it depends on network size and dynamical regime", that the choice of the simpler diffusion model over the slightly more involved jump-diffusion model should not only be based on network size, but also on the dynamical regime under investigation. For statistical comparison, we now added Tables S1 and S2 in the Supplementary Material, where we juxtapose the simulations results of the microscopic, both versions of the mesoscopic, the macroscopic and the RT models for both the fatigue-induced and the fluctuation-induced regimes of hippocampal replay dynamics.

Referee: 2. Which is the advantage of having a state-dependent finite-size noise? In [HT06] the mean-field dynamics is stochastic but is not state-dependent. Here the authors are able to derive from the microscopic dynamics the same equation but with a state-dependent noise. It is indeed modulated not only by the size N but also by the moment Q multiplied by f. Is this an added value by itself? I mean, having a state-dependent noise leads to have qualitatively different collective dynamics compared to what presented in [HT06]? To this purpose it would be interesting to see the time course of $Q(t) * f(h)$ in Figure 2 or 3. A direct comparison with the case of a constant size of the noise (i.e., the σ of [HT06]) would be useful. The result of this investigation could contribute to make more specific the comparison with the model introduced in [HT06] whose related text at page 9-10 should be moved to Discussion section, I would suggest.

Response:
We thank the Referee for this question and take this opportunity to clarify their doubts. First of all, we would like to emphasize that the state-dependent finite-size noise results from the mathematical derivation of the mesoscopic mean-field model. It is hence neither heuristic, i.e. not introduced ad hoc, nor is there an explicit need to specify the noise strength—in our mesoscopic model, there are no free parameters because all derive from the microscopic system.
In order to answer the Referee's question, we can only speculate about the "advantage" of state-dependent over non-state-dependent finite-size noise because it is rather difficult to compare our

mesoscopic model including an explicit link to microscopic dynamics with the model proposed by Holcman and Tsodyks, who introduced their additive noise term ad hoc by saying that "[it] summarizes all uncorrelated sources of noise" [HT06]. In principle, state-dependent noise modulates fluctuations with respect to the current (population) activity, and hence the noise strengths are different in Up vs. Down states. Non-state-dependent noise would assume the same fluctuation strength irrespective of the current state and thereby, in general, lead to smaller residence times in the Down state and longer residence times in the Up state. Single trajectories, however, will not be affected much by state- vs. non-state-dependent noise, since the overall dynamics is guided along the deterministic backbone that is provided by the macroscopic model ($N \to \infty$). Put differently, as long as the noise is small (which can be assumed for medium to reasonably large network sizes), fluctuations (irrespective of whether they are state-dependent or not) will be strong enough to perturb the system away from a macroscopic attractor, but the relaxation dynamics towards the same, or a different, attractor will follow the dynamics of the macroscopic model. On longer time scales, however, the statistics (residence times, inter-population-spike interval distribution, power spectra etc.) will differ significantly.
Moreover, as to the question of what is the "advantage" of the state dependence $Qf(h)$ over $f(h)$ of the finite-size noise, two of us already showed in a previous publication that the second-order mean-field model taking Q explicitly into account yields substantially more accurate descriptions of microscopic networks concerning population spikes and Up-Down transitions, see Figs. 6 and 8 in [Schmutz, Gerstner, Schwalger, J. Math. Neurosci. 2019]. Although qualitative differences on short time scales may be negligible, there are important differences in the respective statistics. In Fig. R1, we compare the time courses of the amplitudes of state- and non-state-dependent noise in the population spikes and Up-Down states regimes. When the state-dependent amplitude $Q * f(h)$ is weighted by the second moment Q of the depression variable x, the peaks of the noise amplitude are reduced not only in size, but also in width, so that strong noise effects only occur in short time windows. This automatically leads to a smaller average noise amplitude $\sigma_Q := \langle Q * f(h) \rangle < \langle f(h) \rangle = \sigma$. The state-dependent noise amplitudes are most of the time below the time averages σ, σ_Q, respectively, which would be used for non-state-dependent noise as, e.g., in [HT06], and which would result in more noise-induced excursions from the Down state.

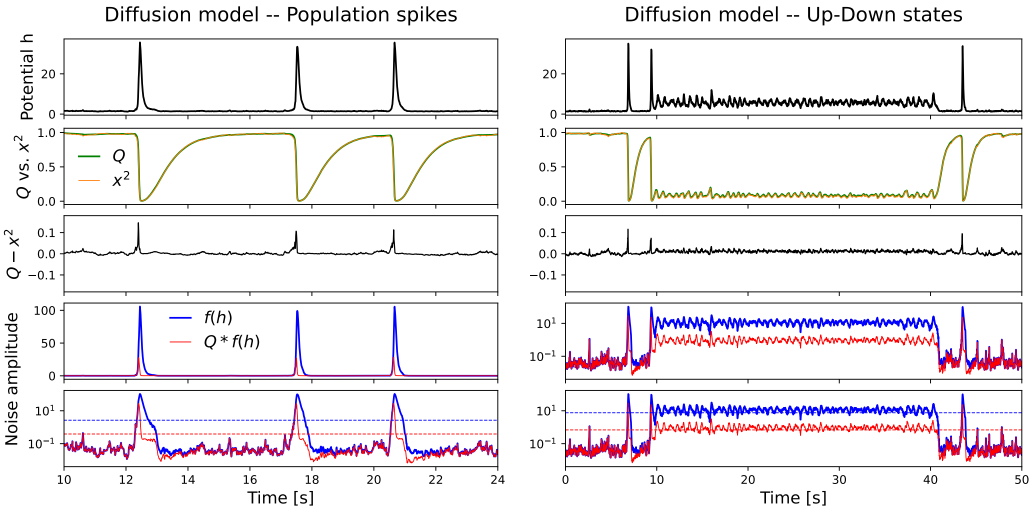

Figure R1: Time courses of the diffusion model Eq. (3) with state-dependent finite-size noise ($N = 100$) in the population spikes (left) and the Up-Down regime (right). From top to bottom: input potential h; the second moment Q (green) compared to the first moment squared x^2 (orange) of the depression variable x; the difference between Q and x^2; amplitude $Q * f(h)$ (blue) of the state-dependent finite-size noise scaled by N compared to the instantaneous rate $f(h)$ (red), their respective means are represented in the semilog plot (bottom) as dashed curves. Model parameters are as in Fig. 3.

Overall, we agree that noise-induced transitions resulting from state- vs. non-state-dependent noise are a very interesting research question per se. This is under current investigation and at this point beyond the scope of the paper, so that these results will be published in future work. We can already remark that it is oftentimes unclear how to properly choose the noise strength of additive, non-state-dependent noise, so that it yields a fair comparison with multiplicative noise, because either choice entails important and distinct consequences for the resulting dynamics.
Last, we followed the Referee's suggestion and moved parts of the text on page 9-10 to the Discussion, see also our response to the Referee's following point.

Referee: 3. Again on the paper length.
I often found the Results subsections having part of the text more suited for the Introduction (see the beginning of "Mesoscopic model for hippocampal replays" page 11) or for the Discussion (see the second half of the subsection "Up-Down dynamics in an excitatory population" pages 10-11). The authors might consider the possibility to make the text leaner and less redundant by moving part of the Results in the Introduction and Discussion sections. About short-term facilitation: is this part of the text really needed?

Response:
We thank the referee for these very valuable suggestions and have tried to make the text leaner and more straightforward. To begin, we did not add new paragraphs to the Introduction to no further increase the complexity of our main messages. We advocate that our theoretical framework and the mesoscopic theory is singularly suited to describe metastable dynamics. In this perspective, we also frame the dynamics of hippocampal replay as one very relevant (and complex) example for metastability. To not get our readers side-tracked from this idea in the Introduction, we keep the (minimal) biological details of hippocampal details as an introductory paragraph to the Results Section on "Mesoscopic model for hippocampal replays". However, we followed the referee's suggestions and moved various parts of the Results subsections to the Discussion:

1. We now discuss in the new Discussion subsection "Diffusion- vs. jump-diffusion model" that the choice of mesoscopic model ought to depend on both the system size and the dynamical regime under investigation (previously on page 9 in Results section "Up-Down dynamics").
2. In the new Discussion subsection "Network size variation resembles...", we draw a hypothetical link between a change in network size and long-term plasticity (previously on page 9-11 in the Results section "Up-Down dynamics").
3. We put the second paragraph of the Results section "Mesoscopic model for hippocampal replays" (page 11) in the Discussion, where we added a new section on "Modelling hippocampal dynamics".

With these changes, we have shortened the Results section by 1.5 pages and we hope that the referees agree that our manuscript is now better streamlined.

Referee: 4. Repetition of waves along the circle.
At page 13 the authors write "a single wave of activity never travels further than once around the circle". This point should be further investigated as it is related to the critical interplay between spatial and temporal scales in the model. Indeed, I guess this happens because the speed of propagation of the wave is so fast that the duration of a NLE is of the same order of the decay time τ_D of the synaptic resources. What happens if the wave speed is lower, less populations are included in the model (smaller M) or a faster recovery from STD (smaller τ_D) is taken into account? What about the stability of the Up state weakened by decreasing the Js? This is another degree of freedom affecting the spatial and temporal extension of the NLE. Furthermore, the authors should consider the fact that noise-driven onset of travelling waves has been studied in model and experiment also in (Capone et al., Cereb. Cortex 2019 – not cited), a similar mechanism which I would suggest to mention at least in the Discussion.

Response:
We thank the Referee for these interesting questions and in particular for pointing us to the reference [Capone et al. 2019], that we now have incorporated in the manuscript both in the Results and Discussion part. We definitely agree with the Referee that a thorough investigation of the interplay between spatial and temporal scales and how this interplay leads to distinct mesoscopic behavior in the ring model is an important line of research. The mathematical difficulty of analyzing the NLE regime of intermittent traveling waves lies in the fact that this dynamical regime appears essentially chaotic. Changing a bifurcation parameter, e.g. the common input μ, drives the dynamics either into a state of ongoing, low-activity traveling waves, or into a homogeneous Down state of global quiescence. This "bifurcation structure" is already present in the macroscopic model used in [RT15]. In this manuscript, we uncovered a noise-induced transition into the dynamical regime where NLEs occur spontaneously from the homogeneous Down state (see Fig. 4). This is a pure finite-size effect because after increasing the number of neurons N per population $1, \ldots, M$ from $N = 50$ to $N = 5000$, the dynamics remained in the Down state and no NLEs occurred. This noise-induced behavior insinuates that our macroscopic model (which is slightly different from the one used in [RT15]) may already exhibit bistability between the NLE regime and the Down state. However, we were not able to tune the initial conditions such that we could realize the NLE regime in the absence of finite-size fluctuations. Another possibility why we did not find this bistability could be that the NLE regime is actually a chaotic transient and that the NLEs emerge along a chaotic saddle. However, even for very long simulation times the NLE regime did not disappear but persisted.
Taken together, an exhaustive analysis of all possible mesoscopic behavior of our ring model is hard to treat analytically. In principle, this can be done numerically. However, we presume that such an analysis will not add significantly to our main message, which is that the bottom-up mesoscopic model reproduces various complex metastable dynamics in microscopic simulations with high accuracy. We think that an in-depth analysis is interesting but beyond the scope of the present paper. We will thus leave the analysis for future work.
Let us now answer the specific question of referee point-by-point:

- "a single wave of activity never travels further than once around the circle"
  We chose model parameters to match the overall dynamics of [RT15], who also observed that the path length of a NLE never exceeded the length of the circle (cf. their Figure 2).
  The mean wave speed (see Fig.4Eiv) is around 12.5 rad/s, so that on average it takes 0.5s to travel around the circle (in line with Fig.4Ei and Eii). The depression time constant is chosen as $\tau_D = 0.8$s, that is of the same order, and thus critically affects the path length as those neurons at the start of the wave are still depressed after 0.5s and the NLE will end here after at most one revolution.

- What happens if the wave speed is lower, less populations are included in the model (smaller M) or a faster recovery from STD (smaller τ_D) is taken into account?
  As can be perceived from Fig.4Eiii, for paths around the full circle, the mean duration is about 0.4s (so even faster than the estimated 0.5s above) and the slowest waves take at most 0.55s to travel around the circle, which is also less than τ_D. For the same reasoning as above, with the selected parameters waves cannot travel further than once around the circle.
  As mentioned above, the dynamical regime featuring NLEs appears chaotic and changing parameters (τ_D or the Js) will quickly drive the dynamics into a different dynamical regime. Therefore, tuning parameters to obtain NLEs traveling farther than once around the circle is non-trivial. A first option would be to enlarge the circle, which can correspond to fewer populations per segment of the circle when keeping the same M. At the same time, enlarging the circle corresponds to reducing the wave speed. In simulations, this does not lead to NLEs traveling farther, but rather to the simultaneous emergence of multiple NLEs (their number depending on the circle size) that are equally distanced around the circle (simulations not shown). Changing only the number of populations M, but not the size of the ring, does not have a large effect on the dynamics as long as the populations are equally spread around the circle. Indeed, the very same behavior of NLEs traveling at most once around the ring is

observed for large $M \gg 100$ and also for M as small as 10; we observed, however, that the fraction of (traveling) NLEs is highly reduced the smaller $M < 20$.
A faster recovery from STD (smaller depression time constant τ_D, while updating U and the Js to maintain the same dynamical regime) enables NLEs to travel further than once around the circle. In simulations of the jump-diffusion model, the dependence of NLEs on τ_D is quite sensitive and leads, beyond a critical depression time constant, to ongoing traveling waves; see Fig. R2 for various values of τ_D. Note also that a faster τ_D leads to slightly faster NLEs. While for $\tau_D = 0.33$ (Fig. R2, top panel) the average time for a full rotation is ~ 0.5s, for $\tau_D = 0.25$ (Fig. R2, bottom panel) the average time for a full rotation reduces to ~ 0.4s.
Another alternative is to make the connectivity matrix J asymmetric, which enforces traveling waves to travel farther than once around the circle, even with higher speed. This was analyzed, e.g., in more detail in [Theodoni et al. eLife 2018], see their Figure 2—main figure as well as figure supplement 7—and Author response image 7; there, the authors also considered $\tau_D = 0.8$s.

- What about the stability of the Up state weakened by decreasing the Js?
We like to emphasize that the NLE regime under investigation does not result from a bistability between homogeneous Up and Down states, but rather from an excitable regime (similar in nature to the one in [Capone et al., Cereb. Cortex 2019]). Therefore, changing the coupling weights will certainly affect the stability of the NLE regime, but not by weakening the Up states (because the Up state does not exist as a global, homogeneous attractor). In the framework of [Capone et al., Cereb. Cortex 2019], presumably the Referee is correct that in individual modules, the (non-)stability of the Up state is weakened further by decreasing the Js.

*Referee: 5. Advantage of the mesoscopic modeling compared to the microscopic one.
In Fig. 4 the equivalence between the two descriptions is further proven but I do not see the advantage of using the former compared to the latter. Computational efficiency? From a theoretical point of view, the phase-plane study is performed on the deterministic version of the model, which from Fig. 5 and Fig. S2 seem to be qualitatively similar to the Romani-Tsodyks model. In other words, what one can do with the mesoscopic dynamics that cannot be done directly resorting to the microscopic model?*

Response:
The critical advantage of the mesoscopic model is that it reduces the the N independent sources of (Poisson) noise (per population) to one source of noise in the case of the pure diffusion model and two sources of noise in the jump-diffusion model (per population). This reduction has two consequences. First, it speeds up simulations: computational time does not grow linearly with the number of neurons as in the microscopic model. Second, from a theoretical point of view, reducing independent neuronal sources of noise to a unique, population-level effective noise is a crucial reduction step for a quantitative description of fluctuation-induced metastable behavior, for which a stochastic description is necessary. For example, the bottom-up description of the noise provides a better understanding of how to model noise in heuristic neural mass and neural field models. For example, noise should be multiplicative instead of additive if it represents finite-size noise, and such differences can strongly affect the statistics of metastable dynamics.

*Referee: Minor Points:
1. Page 4: Why LNP-STP? It should be LNP-STD (short-term depression).*

Response: We have changed the abbreviation LNP-STP to LNP-STD (although, in the Methods section, we provide the mesoscopic equations for general networks of LNP neurons with STP).

Referee: 2. Page 5: "The trajectories hi(t) are càdlàg ...", is it a misprint?

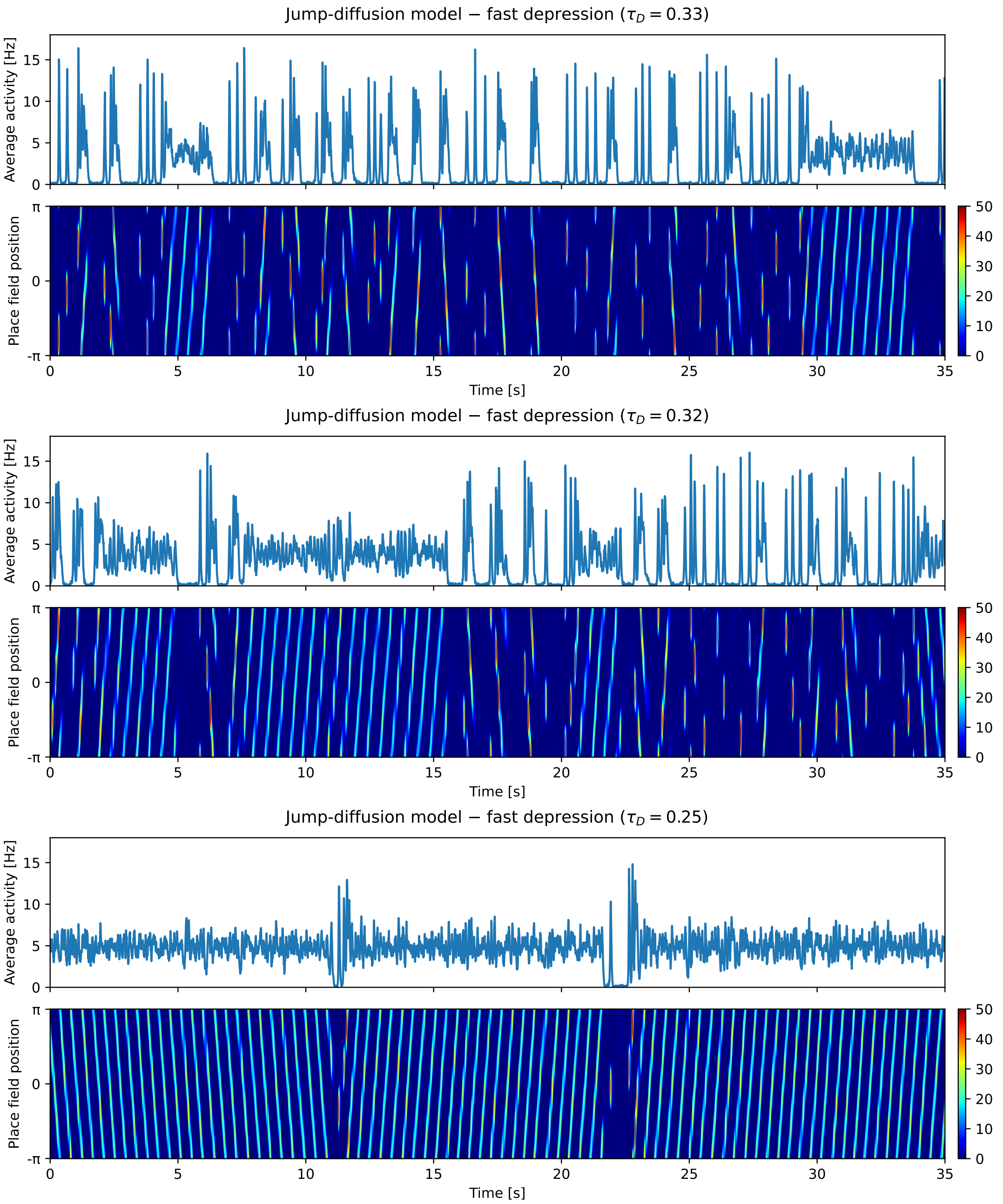

Figure R2: Faster depression $\tau_D \ll 0.8$ leads to NLEs traveling faster and farther than once around the circle in comparison with Fig. 4 from the main text. Model parameters are as in Fig. 4 except for τ_D, see plot titles.

Response: The technical term "càdlàg" has been replaced by "right-continuous".

Referee: 3. Page 5: "It can also be noted that $h_i(t) = h_j(t)$ [... if the] h_i share the same initial condition." I guess this is not correct, as h_i dynamics is determined by a stochastic input related to s_i which is different from neuron to neuron: Am I right?
If this is the case I would suggest to change the title of the subsection "Diffusion approximation for the mesoscopic dynamics with short-term depression" in the Methods, considering the possibility to write "Mean-field approximation for ...".

Response:
The referee were correct if the coupling was not all-to-all. Here, however, we consider global coupling and all neurons receive the same recurrent input $(1/N)\sum_{j=1}^{N} x_j s_j$. Consequently, we keep the title of the subsection as it was.

Referee: 4. Page 23: The statement "conditioned on $h(t^-)$, all the variables ... are independent" should be clarified. Here there is the mean-field approximation at work, as from Eq. (1c) the spike train of the i-th neuron depend on $f(h_i)$ and not on $f(h)$.

Response: The Referee is absolutely right to point out that there were two points of confusion/imprecision in our explanation. First, the conditioning is on the whole past $h_i(t')_{t'<t}$ of all the neurons $i = 1, \ldots, N$. However, since the initial conditions for the h_i are assumed to be identical and the connectivity is all-to-all, conditioning on the microscopic $\{h_i(t')_{t'<t}\}_{i=1}^{N}$ is equivalent to conditioning on the mesoscopic $\{h(t')_{t'<t}\}_{i=1}^{N}$, since $h_i(t')_{t'<t} \equiv h(t')_{t'<t}$. Second, "all the variables [...]" are only *approximately independent* when N is finite; the $\{x_i(t^-)\}_{i=1}^{N}$, even when conditioned on $h(t')_{t'<t}$, are truly independent only when $N \to \infty$. We have clarified/corrected these two points in the Methods section "Diffusion approximation [...]".

Referee: 5. Page 24: "To close the system Eq. (12)", remove "Eq.".

Response: Done.

Referee: 6. Write in the figure captions (Fig. 2-6) that the model parameters can be found in Table 1.

Response: Done.

Referee: 7. Page 8: If I am not wrong, the bistable network in the limit $N->\infty$ is the same as in [HT06]. I would suggest to write it explicitly at the beginning of the "Up-Down dynamics in an excitatory population".

Response:
The referee is right that our bistable network model in the limit $N \to \infty$ is almost the same as the one in [HT06] without noise. We now mention this correspondence explicitly at the beginning of the "Up-Down dynamics in an excitatory population". The main difference, apart from the fluctuations, is the slightly different function $f(h)$, which in our case is a smoothed threshold-linear function, whereas the one considered in [HT06] has a hard threshold.

Referee: 8. The second part of the subsection "Up-Down dynamics in an excitatory population" appears to be more for the Discussion, I would suggest to compress it.

Response: Done, see also our response to the Referee's main point 3.

Referee: 9. Fig. 4 caption: what are the red lines in Panels D and E? Linear regression curves?

Response: Yes, they are linear regression curves. We updated the Figure caption accordingly.

Referee: 10. Fig. 5 and 6: Unit measures in the color bars of panel 5Ci and 6D are not shown: Are they [Hz]?

Response: Yes, we updated the Figures accordingly.

Referee: 11. Page 15: "Assuming that each peak in the average activity corresponds to a SWR". SWR are oscillatory events at specific high frequencies and if I am not wrong, they are not explicitly observed in the model presented in this paper. This statement should than be made more clear and detailed, otherwise I would suggest to remove this reference to SWRs.

Response: The Referee is right that our modelling framework is not capable of producing sharp wave ripples (i.e. high frequency oscillations). We therefore adjusted the respective parts of the manuscript. In particular, we now write at the beginning of the Results section "Mesoscopic model for hippocampal replays":

> Spontaneous replay occurs within so-called sharp waves (SW)—i.e. population bursts of co-active pyramidal cells that give rise to large amplitude extracellular waves— . . .

We do not mention ripples anymore.

Referee: 12. Page 21/22: About the conductance-based models, citations may appear excessively biased. For instance, the sentence "For an extension of the mesoscopic model to a conductance-based description of synaptic input, the interested reader is referred to the discussion of [26]." The authors may consider also to cite the work from the Destexhe's lab (di Volo et al., Neural Comput 2019 – not cited) and (Capone et al., Phys Rev E 2019 – not cited).

Response: We now incorporated the two suggested references.

References

[Aviel and Gerstner, 2006] Aviel, Y. and Gerstner, W. (2006). From spiking neurons to rate models: A cascade model as an approximation to spiking neuron models with refractoriness. *Phys. Rev. E*, 73(5):051908.

[Brinkman et al., 2018] Brinkman, B. A. W., Rieke, F., Shea-Brown, E., and Buice, M. A. (2018). Predicting how and when hidden neurons skew measured synaptic interactions. *PLOS Comput. Biol.*, 14(10):1–40.

[Burak and Fiete, 2012] Burak, Y. and Fiete, I. R. (2012). Fundamental limits on persistent activity in networks of noisy neurons. *Proc. Natl. Acad. Sci. U.S.A.*, 109(43):17645–17650.

[Gerstner et al., 2014] Gerstner, W., Kistler, W. M., Naud, R., and Paninski, L. (2014). *Neuronal Dynamics: From Single Neurons to Networks and Models of Cognition*. Cambridge University Press, Cambridge.

[Hennequin et al., 2018] Hennequin, G., Ahmadian, Y., Rubin, D. B., Lengyel, M., and Miller, K. D. (2018). The dynamical regime of sensory cortex: Stable dynamics around a single stimulus-tuned attractor account for patterns of noise variability. *Neuron*, 98(4):846–860.

[Holcman and Tsodyks, 2006] Holcman, D. and Tsodyks, M. (2006). The emergence of up and down states in cortical networks. *PLoS Comput Biol*, 2(3):e23.

[Ostojic and Brunel, 2011] Ostojic, S. and Brunel, N. (2011). From spiking neuron models to linear-nonlinear models. *PLoS Comput. Biol.*, 7(1):e1001056.

[Romani and Tsodyks, 2015] Romani, S. and Tsodyks, M. (2015). Short-term plasticity based network model of place cells dynamics. *Hippocampus*, 25(1):94–105.
